# Analysis and comparison of the trends in burden of intracerebral hemorrhage in China from 1990 to 2021: results from the 2021 Global Burden of Disease Study

**DOI:** 10.3389/fneur.2025.1578975

**Published:** 2025-08-26

**Authors:** Shuai Han, Zirui Wang, Qiuyue Zheng, Hua Mao, Ju Gao

**Affiliations:** ^1^Institute of Translational Medicine, Medical College, Yangzhou University, Yangzhou, China; ^2^Jiangsu Key Laboratory of Integrated Traditional Chinese and Western Medicine for Prevention and Treatment of Senile Diseases, Yangzhou University, Yangzhou, China; ^3^Northern Jiangsu People's Hospital Affiliated to Yangzhou University, Yangzhou, China; ^4^Peking University People's Hospital, Qingdao, China; ^5^Women and Children's Hospital, Qingdao University, Qingdao, China

**Keywords:** intracerebral hemorrhage, Global Burden of Disease, Joinpoint, age-period-cohort analysis, ARIMA model

## Abstract

**Objectives:**

To analyze temporal trends in the incidence, prevalence, mortality, and disability-adjusted life years (DALYs) of intracerebral hemorrhage (ICH) in China from 1990 to 2021, and to evaluate risk factors and predict future trends.

**Methods:**

Data were extracted from the 2021 Global Burden of Disease Study. Join-point regression was used to estimate average annual percentage changes (AAPC) in ICH incidence and mortality. Age-period-cohort analysis assessed demographic effects, while the autoregressive integrated moving average (ARIMA) model projected ICH burden from 2020 to 2036.

**Results:**

In 2021, China reported 3.12 million ICH cases and 913,023 deaths (68.84 per 100,000). From 1990 to 2021, ICH incidence and mortality significantly declined, with AAPCs of −1.90 and −2.42, respectively. Males exhibited higher rates, and key risk factors included low vegetable intake, hypertension, and smoking. Projections suggest further declines in incidence and mortality to 50.37 and 35.01 per 100,000 by 2036.

**Conclusions:**

Despite declining trends, ICH remains a significant public health concern in China. Targeted preventive strategies focusing on dietary improvements, hypertension management, and air quality enhancement are essential to mitigate its burden.

## 1 Introduction

Intracerebral hemorrhage (ICH) is characterized by bleeding resulting from the rupture of blood vessels within the brain parenchyma (1–3). The onset of ICH constitutes a medical emergency, often associated with high rates of mortality and disability during the acute phase. In China, ICH accounts for ~20%−30% of all stroke cases (4). It predominantly affects the basal ganglia, a region rich in white matter, leading to symptoms such as hemiplegia, partial sensory deficits, ectopic blindness, and other complications (5, 6). Although hemorrhagic stroke occurs less frequently than ischemic stroke, it imposes a significantly higher social and economic burden (7).

The Global Burden of Disease (GBD) study offers comprehensive epidemiological data, providing valuable insights for public health policy through a systematic analysis of diseases and associated risk factors (8–10). The GBD database spans from 1990 to 2021, encompassing data on incidence, prevalence, mortality, and disability-adjusted life years (DALYs). This extensive dataset is crucial for understanding global trends and the determinants of ICH (11–13). In this study, we used the GBD data to characterize the temporal trends of ICH incidence, prevalence, mortality, and DALYs in China from 1990 to 2021, evaluate their age, period and cohort effects, and predict the ICH burden for the next 15 years.

## 2 Materials and methods

### 2.1 Overview

This study analyzes the epidemiology of ICH using annual data on incidence, prevalence, mortality, and DALYs obtained from the GBD database. The study adheres to the Guidelines for Accurate Health Estimates Reporting (14).

### 2.2 Data availability

Data were sourced from the GBD 2021 database, spanning the years 1990 to 2021. ICH was defined according to the International Classification of Diseases, 10th Revision (ICD-10). All data are publicly accessible via the Global Health Data Exchange (GHDx) query tool (https://vizhub.healthdata.org/gbd-results/).

### 2.3 Patient and public involvement

No patients were involved in the research process, including the formulation of the research question, outcome selection, or study design, as the study relied exclusively on publicly available aggregate data.

### 2.4 Statistical analyses

#### 2.4.1 Definitions and methodology

Key terms and definitions from the GBD study are detailed in Supplementary Table 1. Crude disease rates (prevalence, incidence, deaths, and DALYs) serve as fundamental metrics for assessing disease epidemiology. However, variations in population age structure can influence disease burden. To enhance comparability, age-specific weights were applied to crude rates, producing age-standardized rates (ASR). The age-standardized prevalence rate (ASPR), incidence rate (ASIR), mortality rate (ASMR), and DALYs rate (ASDR) were subsequently used to estimate the burden of ICH.

#### 2.4.2 Joinpoint regression analysis

Joinpoint regression analysis, using a series of linear statistical models, was applied to evaluate temporal trends in the burden of ICH (15). This method, employing least squares estimation, avoids the bias inherent in traditional linear trend analyses by identifying significant turning points in trends (16). The model minimizes the sum of squared residuals between predicted and observed values. Joinpoint software was used to calculate the average annual percentage change (AAPC), with statistical significance assessed at *p* < 0.05.

#### 2.4.3 Age-period-cohort analysis

The age-period-cohort (APC) model was used to assess the effects of age, period, and cohort on ICH outcomes (17). The age effect evaluates risk across different ages, the period effect examines temporal changes across all age groups, and the cohort effect analyzes outcomes within specific birth cohorts. The log-linear regression model is expressed as: log(Y_i_) = log(Y_i_) = μ + α ^*^ age_i_ + β ^*^ period_i_ + γ ^*^ cohort_i_ + ε, where Y_i_ represents the prevalence or mortality rate, μ is the intercept, and ε denotes the residual. Coefficients α, β, and γ correspond to age, period, and cohort effects, respectively (18).

#### 2.4.4 Decomposition analysis

Das Gupta's decomposition method was employed to quantify the contributions of age structure, population growth, and epidemiological changes to the ICH burden in China.

#### 2.4.5 Autoregressive integrated moving average (ARIMA) model

The ARIMA model integrates autoregressive (AR) and moving average (MA) components to analyze time-dependent data (19). It assumes that the series is stationary, stochastic, and has a zero mean, enabling predictions based on historical values. This model captures autocorrelation within the data and facilitates robust trend forecasting.

All analyses and visualizations were conducted using R software (version 4.4.2), with statistical significance set at *p* < 0.05.

## 3 Results

### 3.1 Description of the burden of ICH

Between 1990 and 2021, the prevalence of ICH in China increased from 3,115,040 to 4,385,240 cases, reflecting a growth of 40.77%. Despite this rise, the ASPR decreased from 308.41 to 222.11 per 100,000 population, with an AAPC of −1.07%. The incidence of ICH rose from 774,012 to 1,173,288 cases, marking a 51.59% increase, while the ASIR declined from 108.93 to 61.15 per 100,000, with an AAPC of −1.90%. Mortality associated with ICH grew from 913,023 to 1,322,893 deaths (44.90% increase), but the ASMR significantly dropped from 139.67 to 68.84 per 100,000, with an AAPC of −2.42%. Additionally, DALYs attributed to ICH increased from 22,779,117 to 27,463,746, while the ASDR decreased from 2,830.02 to 1,351.55 per 100,000, with an AAPC of −2.48%. The data indicate that males bear a higher disease burden than females across various age groups (Supplementary Figure 1, Table 1).

**Table 1:** All-age and age-standardized rates of incidence, prevalence, mortality, and DALYs with AAPC for ICH in China (1990 and 2021).

**Measure**	**1990**		**2021**		**1990-2021 AAPC**
	**All-age cases**	**Age-standardized rates per 100,000 people**	**All-age cases**	**Age-standardized rates per 100,000 people**	
	**n (95% UI)**	**n (95% UI)**	**n (95% UI)**	**n (95% UI)**	**n (95% CI)**
Prevalence	3,115,040 (2,764,294, 3,518,252)	308.41 (274.49, 348.29)	4,385,240 (3,892,101, 4,906,565)	222.11 (200.09, 246.48)	−1.07 (−1.12, −1.02)
Incidence	774,012 (644,709, 896,197)	108.93 (91.66, 124.93)	1,173,288 (1,003,993, 1,330,455)	61.15 (52.98, 69.06)	−1.90 (−1.97, −1.83)
Deaths	913,023 (784,398, 1,064,534)	139.67 (121.09, 162.03)	1,322,893 (1,108,046, 1,567,711)	68.84 (57.61, 81.17)	−2.42 (−2.06, −1.00)
DALYs	22,779,117 (19,630,525, 26,510,841)	2,830.02 (2,441.76, 3,281.07)	27,463,746 (22,839,243, 32,676,709)	1,351.55 (1,129.11, 1,600.86)	−2.48 (−2.21, −1.00)

AAPC, average annual percentage change; UI, uncertainty interval; CI, confidence interval.

Age-standardized years lived with disability (YLDs), and years of life lost (YLLs) rates showed similar trends by sex and age group. The all-age numbers and age-standardized rates for males and females are presented in Supplementary Tables 2, 3. It is clear that males have a higher disease burden than females.

### 3.2 Gender disparities in the burden of ICH in different age groups

Supplementary Figures 2, 3 present the incidence, prevalence, mortality, and DALYs of ICH across age groups for males and females in China in 2021. Prevalence increased sharply between ages 40 and 59, peaking at 55–59 years, and remained stable from 60 to 74 years, with males consistently showing higher case numbers than females across most age groups. Incidence peaked at 75–79 years for both sexes, with a significant rise after age 65, and males again surpassed females except in the youngest and oldest age groups. Mortality and DALYs followed similar patterns, with both metrics peaking at 75–79 years and increasing dramatically after age 75. Males exhibited higher mortality and DALYs than females across all age groups, except for the 0–5 group.

**Figure 1:**
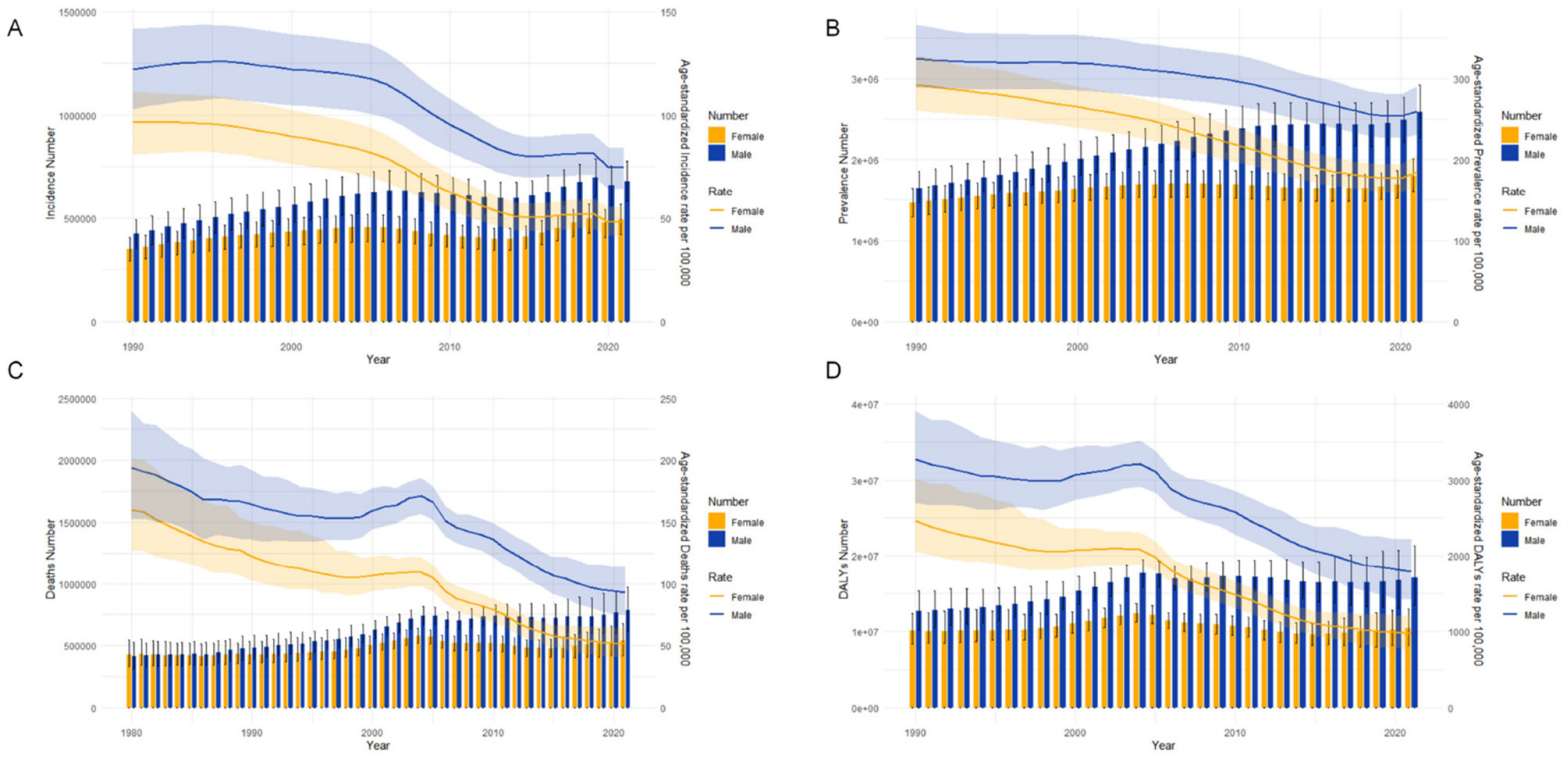
Comparison of age-specific and age-standardized rates for incidence, prevalence, mortality, and disability-adjusted life years (DALYs) of intracerebral hemorrhage in males and females in China from 1990 to 2021. **(A)** Incident cases and age-standardized incidence rate (ASIR); **(B)** Prevalent cases and age-standardized prevalence rate (ASPR); **(C)** Death cases and age-standardized mortality rate (ASMR); **(D)** DALYs counts and age-standardized DALY rate (ASDR). Bar charts represent counts, while lines represent age-standardized rates.

Figure 1 compares the disease burden and ASRs of ICH in males and females across all ages in China from 1990 to 2021. In Figure 1A, the ASIR peaked in 1990, showing the largest gender disparity, with higher rates in males. Over time, the ASIR declined steadily, and the gender gap narrowed. Similarly, the ASPR decreased consistently from 1990 to 2021, remaining higher in males than females throughout the period (Figure 1B). Figure 1C highlights that, in 2021, males exhibited a significantly higher ASMR compared to females, although overall ASMR showed a downward trend over the years. Likewise, the ASDR mirrored the ASMR trends, with higher values in males and a general decline over time (Figure 1D).

### 3.3 Joinpoint regression analysis of the burden of ICH

**Figure 2:**
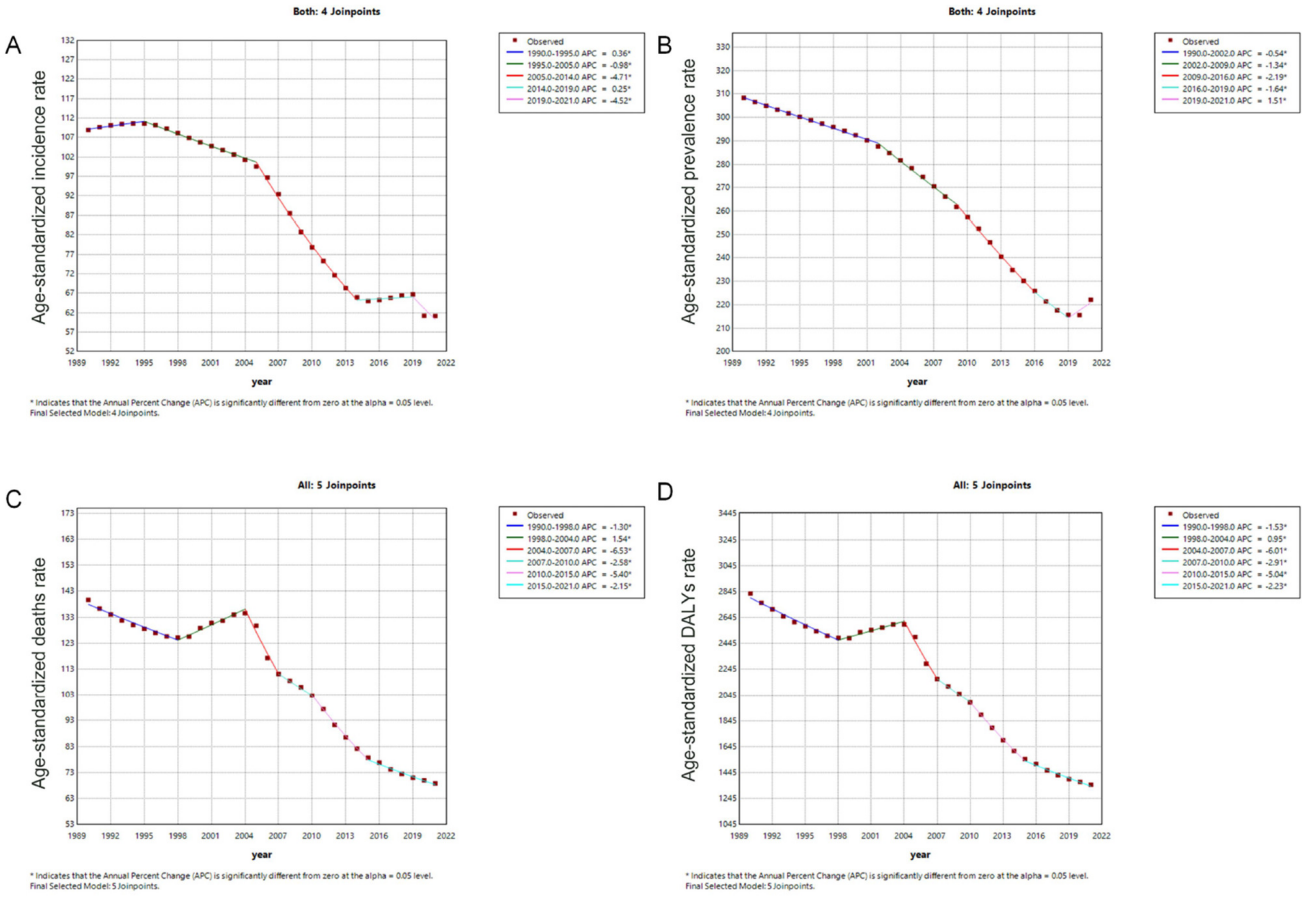
Trends in age-standardized rates (per 100,000 population) for intracerebral hemorrhage in China from 1990 to 2021. **(A)** Age-standardized incidence rate; **(B)** Age-standardized prevalence rate; **(C)** Age-standardized mortality rate; **(D)** Age-standardized DALYs rate. DALYs, disability-adjusted life years.

Figure 2 presents the Joinpoint regression analysis of ASIR, ASPR, ASMR, and ASDR for ICH in China from 1990 to 2021. Overall, the annual percentage change (APC) for ASIR and ASPR demonstrated a consistent decline. A significant reduction in ASIR occurred between 2005 and 2014 [APC = −4.71 (−4.79, −4.63), *p* < 0.05], while ASPR showed a marked decrease from 2009 to 2016 [APC = −2.19 (−2.26, −2.11), *p* < 0.05]. For ASMR and ASDR, an initial decline was observed from 1990 to 1998 [ASMR: APC = −1.51 (−1.89, −0.60); ASDR: APC = −1.69 (−3.37, −0.83), *p* < 0.05], followed by a temporary increase from 1998 to 2004. Subsequently, a significant decrease was noted from 2004 to 2007 [ASMR: APC = −7.47 (−9.59, −3.58); ASDR: APC = −6.72 (−9.29, −4.72), *p* < 0.05] (Supplementary Table 4).

### 3.4 Age, period and cohort effects on ICH incidence and mortality

**Figure 3:**
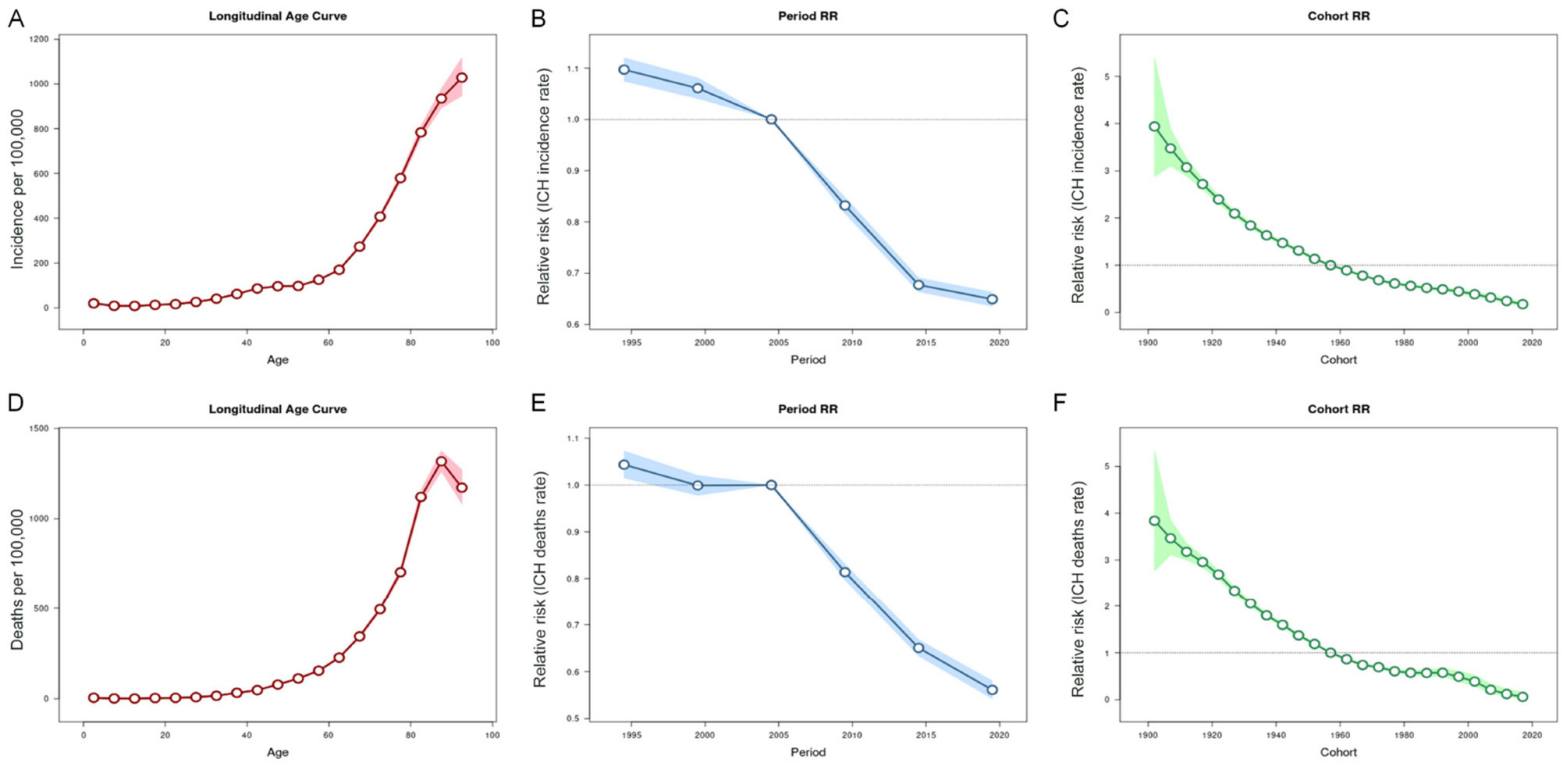
Age, period and cohort effects on intracerebral hemorrhage (ICH) incidence and mortality rate in China during 1990-2021. **(A–C)** Represent the 95% CI of age, period and cohort effects for ICH incidence; **(D–F)** Represent the 95% CI of age, period and cohort effects for ICH mortality rate.

Figure 3 depicts the age-period-cohort effects on ICH incidence and mortality rates. ICH incidence increased from age 27.5, peaking at age 92.5. Period-based trends revealed a steady decline, with relative risk (RR) values decreasing from 1.10 (95% CI: 1.08–1.12) in 1995 to 0.65 (95% CI: 0.64–0.66) in 2019. Cohort effects showed significantly higher risk in early birth cohorts [RR_cohort_ (1902) = 3.94, 95% CI: 2.88–5.39], which progressively decreased in recent cohorts [RR_cohort_ (2017) = 0.17, 95% CI: 0.13–0.23]. Age-specific mortality rates from ICH followed an exponential increase, with RR values rising gradually before age 42.5 and accelerating thereafter, peaking near age 87.5 before a slight decline at 92.5. Period-based mortality trends shifted around 2004, with the RR stabilizing at 1.0 and decreasing thereafter [RR_period_ (2019) = 0.56, 95% CI: 0.54–0.58]. Similar to incidence, early birth cohorts had a greater impact on mortality, with RR values declining from 3.84 in the 1902 cohort to 0.06 in the 2017 cohort (Supplementary Table 5).

### 3.5 Decomposition analysis of ICH

Decomposition analyses revealed a substantial increase in the burden of ICH from 1990 to 2021. By 2021, there were over 4.38 million prevalent cases (a 140.78% increase), 1.17 million incident cases (a 151.59% increase), 1.32 million deaths (a 144.89% increase), and 27.46 million DALYs due to ICH (a 120.57% increase) (Supplementary Table 6). The primary drivers of these increases were population aging and epidemiological changes. Aging contributed to 126.55%, 245.32%, 280.61%, and 568.23% increases in prevalence, incidence, deaths, and DALYs, respectively, over the past 30 years. Epidemiological changes, in contrast, reduced these indicators by −117.23%, −249.75%, −280.35%, and −708.48%, respectively. Notably, compared to males, females exhibited a greater impact of epidemiological changes on prevalence, deaths, and DALYs (Supplementary Figure 4).

### 3.6 Risk factors

Key risk factors contributing to ICH-related DALYs and deaths in 2021 included a diet low in vegetables, high systolic blood pressure, particulate matter pollution, air pollution, and smoking. The impact of these factors varied by sex: smoking, tobacco, and alcohol consumption had a greater influence on DALYs in males, while hypertension and particulate matter pollution were significant risk factors for both sexes. Addressing these modifiable risk factors through targeted interventions could significantly reduce the burden of ICH (Supplementary Tables 7, 8).

### 3.7 Prediction of ICH ASIR and ASDR in the next fifteen years

**Figure 4:**
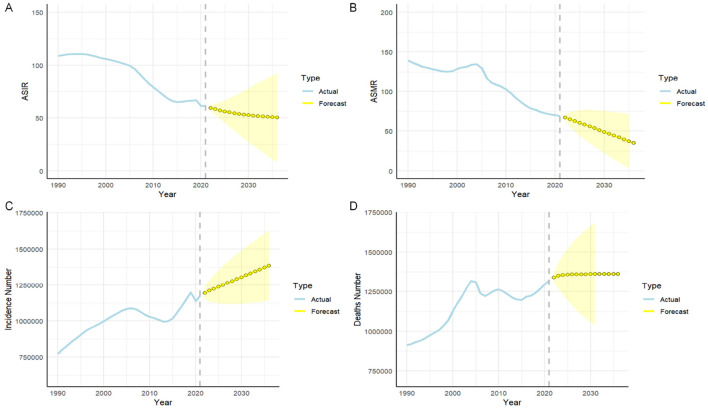
Predicted trends of intracerebral hemorrhage (ICH) prevalence and mortality rate in China over the next fifteen years (2022-2036). **(A)** Age-standardized incidence rate; **(B)** Age-standardized mortality rate; **(C)** The number of incidence; **(D)** The number of mortality. Blue line represent the true trend of ICH prevalence and mortality rate during 1990–2021; yellow dot lines and shaded regions represent the predicted trend and its 95% CI.

The ARIMA model was applied to forecast trends in ICH ASIR and ASDR over the next 15 years. The optimal model for ASIR was identified as ARIMA (1,1,1) with an AIC of 115.82, while ARIMA (0,2,0) was selected for ASMR with an AIC of 139.76. Projections indicate that by 2036, the ASIR and ASMR will stabilize at ~50.37 per 100,000 and 30.01 per 10,000, respectively. The incidence of ICH is expected to increase from 1,193,992 cases in 2022 to 1,385,305 cases in 2036, with mortality rising from 1,340,767 to 1,360,969 cases over the same period (Figure 4).

## 4 Discussion

This study provides a comprehensive analysis of the burden of ICH in China using GBD data from 1990 to 2021, revealing a decline in ASPR, ASIR, ASMR, and ASDR over recent decades, consistent with global trends (20). By extending data to 2021, it offers an updated overview of epidemiological trends and risk factors, addressing the gap from 2019 to 2021. This update is crucial as the COVID-19 pandemic redirected healthcare resources, highlighting the need for current data on other diseases. The inclusion of recent data enables policymakers to identify emerging trends and implement more targeted prevention strategies.

The ASIR of ICH in China has significantly declined since 1990, yet its prevalence remains higher than the global average (21). Between 1990 and 2021, absolute ICH cases in China increased by 40.77% (from 3.12 to 4.39 million), driven by population aging. In 2021, ICH caused 1.32 million deaths in China, making it the second leading cause of death, with a mortality rate exceeding earlier GBD stroke estimates, likely due to differences in data sources, reporting methods, and the impact of COVID-19 (5, 22). Males experienced higher mortality and DALYs, indicating a greater burden of long-term disability (23, 24). Age, period, and cohort analyses revealed that ICH incidence rises sharply after age 27.5, peaking at 92.5, while period effects showed a decline in relative risk (RR) from 1.10 in 1995 to 0.65 in 2019, reflecting healthcare improvements. Cohort effects indicated higher risks in earlier cohorts (RR = 3.94 for 1902) and lower risks in recent ones (RR = 0.17 for 2017), suggesting lifestyle changes and medical advancements have reduced ICH risk. Mortality peaked at age 87.5 but has declined since 2004, reflecting improved acute care and survival rates. Decomposition analysis from 1990 to 2021 shows that population aging was the primary driver of increased ICH cases, deaths, and DALYs, significantly contributing to the rising burden (25, 26). In contrast, advancements in healthcare and stroke management alleviated the burden, reducing prevalence, incidence, mortality, and DALYs, with females benefiting more than males (27, 28).

Key risk factors for ICH include hypertension, a low vegetable diet, smoking, air pollution, and exposure to particulate matter, with hypertension being the most significant modifiable factor (29, 30). The ongoing impact of particulate matter pollution and rising smoking rates in 2021 underscore the need for stronger environmental management and tobacco control policies. Our findings indicate that smoking and heavy alcohol consumption are more prevalent among men, significantly increasing their ICH risk—studies show a threefold increase for men compared to women. Hypertension affects both genders differently; men experience higher rates earlier, while women become vulnerable post-menopause, highlighting the need for tailored management strategies (31). Environmental factors like air pollution may also disproportionately affect women, necessitating targeted interventions. We recommend initiatives to reduce tobacco and alcohol use in men, adapt hypertension screening for women, and implement community workshops that include gender-sensitive healthcare training. These insights clarify gender-specific impacts on ICH risk factors and inform effective public health strategies.

ARIMA model projections indicate that while ASIR and ASMR for ICH may stabilize over the next 15 years, the overall burden will rise due to population aging, driving increases in cases and mortality despite stable rates per 100,000 population. This trend highlights the urgent need for enhanced prevention and treatment strategies, particularly for the aging population. Long-term planning for healthcare resources, improved hypertension management, smoking reduction, and better dietary habits are critical to mitigating the growing ICH burden.

This study provides a comprehensive analysis of the ICH burden in China using GBD data from 1990 to 2021, revealing declining ASIR, ASPR, ASMR, and ASDR, though continued efforts are needed. Public health policies should focus on hypertension management, smoking control, environmental improvements, and expanded healthcare coverage to reduce ICH incidence and mortality. While the study's strength lies in its large-scale data and long-term trend analysis, limitations in statistical modeling highlight the need for improved data and methods in future research to better inform prevention and control strategies.

## 5 Conclusion

This study revealed significant declines in ASIR, ASPR, ASMR, and ASDR of ICH in China from 1990 to 2021, with this trend expected to continue until 2036. However, ICH mortality rises exponentially after age 42.5, with high-risk groups including those with hypertension, a low vegetable diet, and air pollution exposure. Targeted public health strategies are crucial to reducing the ICH burden and preventing avoidable deaths.

## Data Availability

The original contributions presented in the study are included in the article/Supplementary material, further inquiries can be directed to the corresponding authors.
