# Supplementary material for: Analysis and comparison of the trends in burden of intracerebral hemorrhage in China from 1990 to 2021: results from the 2021 Global Burden of Disease Study
Source: Front Neurol. 2025 Aug 26;16:1578975. doi: 10.3389/fneur.2025.1578975 (PMC12417145; doi:10.3389/fneur.2025.1578975)
Supplement: Supplementary file 1 [file Table_1.docx]

**Supplementary Figure 1 -** Crude and age-standardised incidence, and mortality rate of intracerebral hemorrhage per 100 000 population in 2021, by country. (A) Crude incidence rate; (B) Crude mortality rate (CMR); (C) Age-standardised incident rate (ASIR); (D) Age-standardised death rate (ASMR).

**Supplementary Figure 2 -** Gender-specific comparison of the number of prevalence, incidence, mortality, and disability-adjusted life years (DALYs) of intracerebral hemorrhage across different age groups in China, 2021. (A) Incidence; (B) Prevalence; (C) Mortality; (D) DALYs.

**Supplementary Figure 3 -** Gender-specific comparison of the rate of prevalence, incidence, mortality, and disability-adjusted life years (DALYs) of intracerebral hemorrhage across different age groups in China, 2021. (A) Incidence; (B) Prevalence; (C) Mortality; (D) DALY rate.

**Supplementary Figure 4 -** Decomposition analysis of intracerebral hemorrhage from 1990 to 2021. (A) Prevalence, (B) Incidence, (C) Deaths, and (D) DALYs. The black dot represents the overall change value of population growth, aging, and epidemiological change. DALYs, disability-adjusted life years.
